# Intrascrotal lipoblastoma: report of a case and the review of literature

**DOI:** 10.1186/s40792-016-0160-7

**Published:** 2016-04-09

**Authors:** Keigo Yada, Hiroki Ishibashi, Hiroki Mori, Mitsuo Shimada

**Affiliations:** Department of Surgery, The University of Tokushima, 3-18-15 Kuramoto-cho, Tokushima City, Tokushima 770-8503 Japan

**Keywords:** Intrascrotal lipoblastoma, Scrotal incision, Preoperative diagnosis

## Abstract

Intrascrotal lipoblastoma is a rare pediatric benign soft tissue neoplasm, and only 11 cases have been reported. The accurate preoperative diagnosis is difficult because of its rarelity and the similarity with the other soft tissue tumors. Among them, accurate preoperative diagnosis had been made in only one case. Thus, almost all of the cases had required inguinal mass excision (and orchidectomy in one case). In this paper, we discuss the accurate preoperative diagnosis of intrascrotal lipoblastoma and subsequent simple tumorectomy via minimal invasive scrotal skin incision, in 1-year-old boy. On physical examination, intrascrotal extra-testicular lobulated mass was palpated on the right scrotum. An ultrasonography revealed the well-circumscribed, iso-echoic, scant blood-flow, and lobulated tumors with each lobules of 1 to 4 cm in diameter, and the tumor located outside of the tunica vaginalis testis. The serum values of alpha-fetoprotein (AFP) and beta-human chorionic gonadotropin (b-hCG) were within normal limit. The preoperative diagnosis of intrascrotal lipoblastoma was made, and the mass was excised via minimal scrotal incision. The right testicle and epididymis were normal. The lesion consisted of the distinct two lobulated tumors, and microscopic examination confirmed the diagnosis of intrascrotal lipoblastoma. The postoperative course was uneventful without evidence of recurrence. A rare intrascrotal lipoblastoma is seldom made accurate preoperative diagnosis; however, the accurate preoperative suspicion of this tumor leads to the minimal invasive tumorectomy via scrotal skin incision and favorable postoperative recovery without recurrence.

## Background

In the first two decades of life, adipose tumors are relatively rare, comprising about 6 % of soft tissue neoplasms. About 60 % of these are simple lipomas or variants, and up to 30 % are lipoblastomas [1]. It is found most commonly in the trunk or upper and lower extremities as a painless nodule or mass [1]. Less common sites of involvement include the head and neck area [2], mediastinum [3], mesentery [4], omentum [5], retroperitoneum [6], and scrotum [7–16]. The accurate preoperative diagnosis is difficult because of its rarelity and the similarity with the other soft tissue tumors [17]. Previously, 11 cases of intrascrotal lipoblastoma have been reported [7–16]. Among them, accurate preoperative diagnosis had been made in only one case [14], and an another case had been underwent orchidectomy due to the preoperative suspicion of paratesticulsr rhabdomyosarcoma [13]. This report describes a case in which accurate preoperative diagnosis of this rare tumor z procedure (e.g., inguinal incision and orchidectomy) and to result in the favorable outcomes.

## Case presentation

**Fig. 1:**
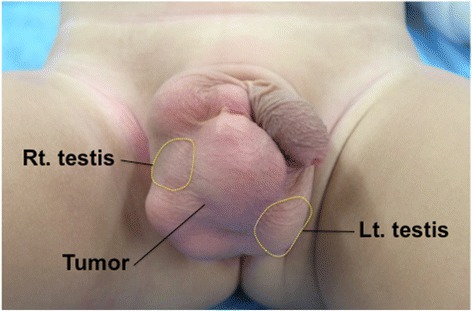
The preoperative finding of the scrotum. The tumors were apart from the bilateral testes

**Fig. 2:**
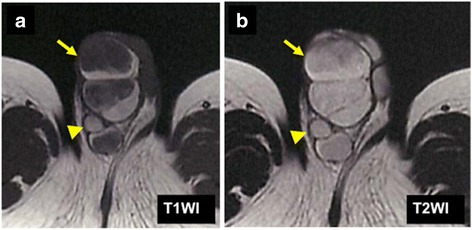
The MRI findings. **a** T1-weighted image showed the mosaic pattern (e.g., T1-low lesions and T1-high lesions were co-existed). **b** All of the tumor revealed the high intensity on T2-weighted image. (*Arrow heads* indicate right testis.)

**Fig. 3:**
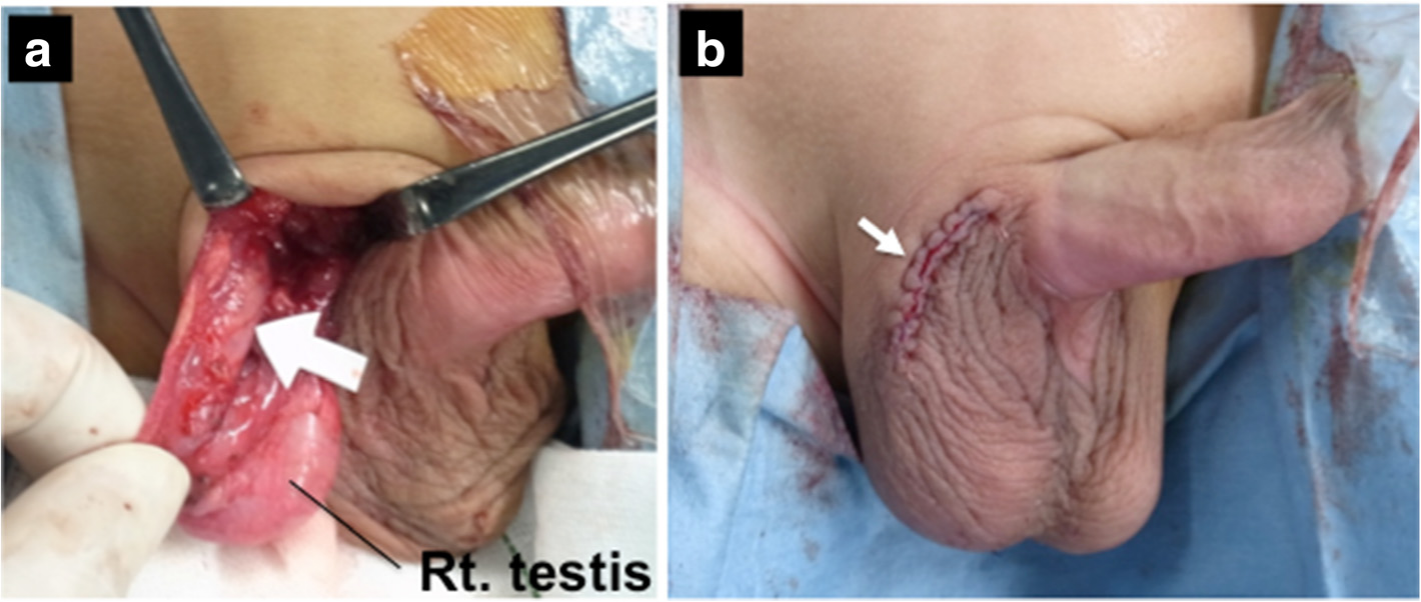
Operative findings of the scrotum. **a** The lesion located out of the cord and was independent of the normal right testicle and of the processus vaginalis. (*White arrow* indicates the tumor location.) **b** The mass was excised via the minimal scrotal incision

**Fig. 4:**
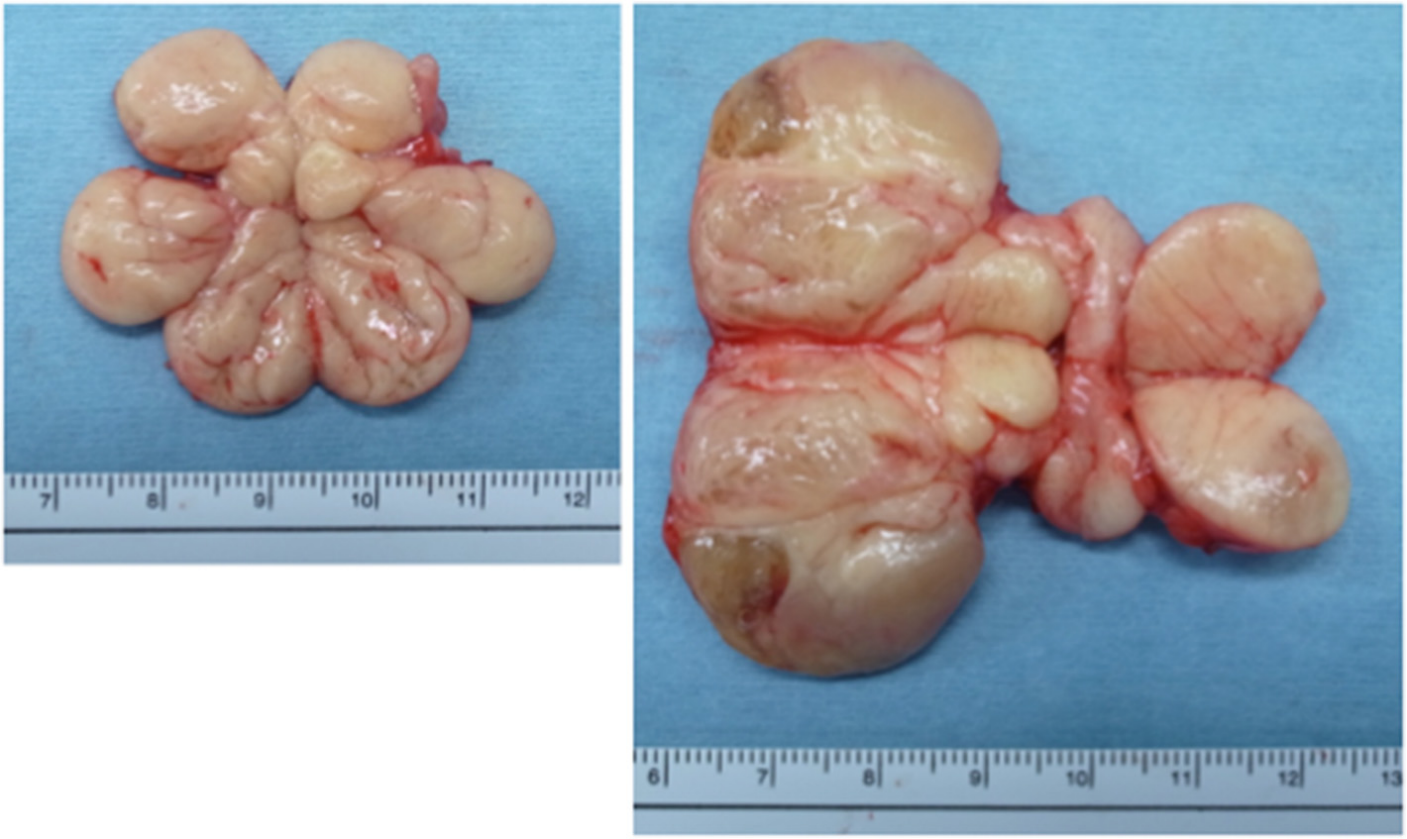
The cut surface of the specimens. The lesion consisted of the distinct two lobulated and well-circumscribed tumors with diameter of 6 cm × 4 cm × 3 cm and 3.5 cm × 2 cm × 2 cm, respectively

A 1-year and 7-month-old boy was taken to his pediatrician after his family members noticed a right swollen scrotum. An intrascrotal tumor was suspected, and he was referred to our pediatric surgery department. On physical examination, a 7-cm lobulated mass was palpated on the right scrotum, apart from the normal bilateral testes (Fig. 1). The rubbery-hard tumor had smooth surface and was mobile, suggesting benign pathology. An ultrasonography revealed the well-circumscribed, hyper-echoic, scant blood-flow, and lobulated tumors with each lobules of 1 to 4 cm in diameter, and the tumor is located outside of the tunica vaginalis testis. A contrast computed tomography (CT) scan showed the less enhancement of the tumors. All of the tumors revealed the high intensity on magnetic resonance imaging (MRI) T2-weighted image, but T1-weighted image showed the mosaic pattern (e.g., T1-low lesions and T1-high lesions were co-existed) (Fig. 2a, b). The serum values of alpha-fetoprotein (AFP) and beta-human chorionic gonadotropin (b-hcg) were within normal limit. We considered that the most likely preoperative diagnosis is the intrascrotal lipoblastoma, as the previously reported cases [8, 10, 11, 14, 15] had the quite similar features (e.g., a young child with well-circumscribed and lobulated intrascrotal lipoid mass apart from the testes or the epididymis) with our case. The other differential diagnoses were rhabdomyosarcoma, lipoma, and liposarcoma. The mass was excised by scrotal incision (Fig. 3b). The lesion developed out of the cord and was independent of the right testicle and of the processus vaginalis (Fig. 3a, b). The right testicle was normal (Fig. 3a). The lesion consisted of the distinct two lobulated and well-circumscribed tumors with diameters of 6 cm × 4 cm × 3 cm and 3.5 cm × 2 cm × 2 cm, respectively (Fig. 4).

**Fig. 5:**
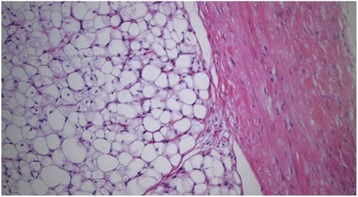
Pathological analysis. Microscopically, both tumors were multilobulated tumors of adipose tissue and were surrounded by the capsule composed of loose connective tissue. The both tumors were diagnosed as lipoblastoma (hematoxylin-eosin stain, ×100)

Microscopically, both tumors were multilobulated tumors of adipose tissue and were surrounded by the capsule composed of loose connective tissue. Lobules were separated by fibrous septa and were composed of mature adipocytes and vacuolated lipoblasts showing varying degrees of differentiation. Both tumors were diagnosed as lipoblastoma (Fig. 5).

The postoperative course was uneventful, and the patient discharged home on postoperative day 1. The patient has been followed up for 8 months, without evidence of recurrence.

### Discussion

Lipoblastoma, a relatively rare tumor of embryonal fat, is characterized by its benign nature, early presentation (90 % <3 years) [18], male predominance of 3:1 [19], and rapid growth. Although it is found most commonly in the trunk or upper and lower extremities [1], the involvement of the other lesion (e.g., head/neck [2], mediastinum [3], mesentery [4], omentum [5], retroperitoneum, and scrotum (Table 1) [7–16]) has been reported. The differential diagnoses of pediatric intrascrotal paratesticular tumor include benign tumors (e.g., lipoma, lipoblastoma, leimomyoma, and hemangioma) and malignant tumors (e.g., rhabdomyosarcoma, liposarcoma, and melanotic neuroectodermal tumor of infancy (MNTI)) [15, 20]. Of these, leiomyoma and MNTI generally originate from the epididymis, and hemangioma has the much vasculature. So at first, we could deny these three tumors. Rhabdomyosarcoma is the most common paratesticular malignancies in pediatric population [21], and it arises from the tunicae of the testis, epididymis, and spermatic cord. But, rhabdomyosarcoma commonly occurs in older children with median age of 7 years old [22] and reveals heterogeneous echogenicity and increased blood flow in ultrasonography (US) [23]. Liposarcoma lacks lobulated shape, and occurrence in pediatric population is extremely rare [20]. Also, lower intensity of T1-weighted MRI image has been noted with lipoblastoma compared with lipoma [24], likely because of increased cellularity. In our case, the intratumoral mosaic pattern (e.g., T1-low lesions and T1-high lesions were co-existed) on T1-weighted image suggested the lipoblastoma, rather than a lipoma. Furthermore, lipoblastoma has the following distinguishable characteristics: I: commonly involves in patients younger than 3 years old, predominantly in male by 3:1 and II: well-circumscribed, lobulated, hypovascular fatty tumor. Thus, we could make accurate preoperative diagnosis of lipoblastoma. Our case is the second case in which accurate preoperative diagnosis of lipoblastoma could be made (Table 1). However, the differential diagnosis of intrascrotal tumor in older patients might be more complicated, because the origin of the testicular or paratesticular might sometimes unclear. The testicular lesion includes teratoma, seminoma, or non-neoplastic lesion (e.g., hydrocele or inguinal hernia) [20].

**Table 1:** Summary of cases of intrascrotal lipoblastoma in the literature

Patient	Authors	Year	Age	Side	AFP (ng/ml)	*β* hCG (UI/L)	Preoperative image findings	Preoperative diagnosis	Approach	Treatment	Size of tumor (cm × cm × cm)
1	Arda et al.	1993	15 months	Left	N.A	N.A	N.A	N.A	Inguinal	Mass excision	6.5 × 4 × 3
2	Turner et al.	1998	9 months	Left	11	1.8	US: lobulated solid lesion with well-defined margin.	N.A	Inguinal	Mass excision	5 × 2.5 × 1.5
3	Chun	2001	18 months	N.A	N.A	N.A	N.A	N.A	N.A	N.A	2.3 × 2 × 1.3
4	Somers et al.	2004	7 months	Left	N.A	N.A	US: heterogeneous mass consisting of small cystic areas admixed with solid components.	N.A	Inguinal	Mass excision	14 × 14 × 8^a^
5	Dy et al.	2007	4 years	N.A.	N.A	N.A	N.A	N.A	Inguinal	Mass excision	3.5 × 2 × 2
6	Del Sordo et al.	2007	4 years	Right	N.A	N.A	N.A	N.A	Inguinal	Mass excision	2 × 1.2 × 0.8
7	Robb et al.	2010	10 months	Left	N	N	US: echogenic mass with good vascularity, separate to the testis.	RMS	Inguinal	Orchidectomy and Mass excision	3 × 2.5 × 1.5
8	Kamel et al.	2011	4 months	Right	N.A	N.A	US and CT: large fatty tumor.	Lipoblastoma	Inguinal	Mass excision	10 × 9 × 7
9	Nakib et al.	2013	10 years	Right	N	N	US: a hyper-echoic lesion above the upper pole of the testicle.	N.A	N.A	Mass excision	5 × 5 × 1.5
10	Eyssartier et al.	2013	15 months	Left	17	4	US: echogenic solid lesion with well-defined margin.	N.A	Inguinal	Mass excision	2 × 1.5 × 1
11	Eyssartier et al.	2013	16 months	Right	7.8	<1	US: an echogenic mass.	N.A	Inguinal	Mass excision	1.5 × 1.5 × 2
12	Present case	2015	19 months	Right	6.0	225	US: hyper-echoic lobulated mass with well-circumscribed margin. CT: less enhanced mass. MRI: TI high/low, T2 high.	Lipoblastoma	Scrotal	Mass excision	6 × 4 × 3, 3.5 × 2 × 2

*N.A* not available; *N* normal; *US* ultrasonography; *RMS* rhabdomyosarcoma

^a^In this case, the other two residual lesions were removed postoperatively

Regarding the surgical treatment, malignant lesions require the gold-standard surgical approach of radical inguinal orchidectomy [25]. On the other hand, standard treatment of the lipoblastoma is the complete resection of the tumor [17], to avoid recurrence. In fact, among the previously reported cases of intrascrotal lipoblastoma (Table 1), almost all of the cases had been performed inguinal mass excision (and orchidectomy in one case). Our case is the first case in which simple tumorectomy was performed via minimal scrotal skin incision. We consider that if the adequate preoperative suspicion of lipoblastoma could be made, we can avoid the invasive procedure (inguinal exploration or orchidectomy). In our case, accurate preoperative diagnosis of this rare tumor led to the simple tumor resection via minimal scrotal skin incision and favorable postoperative recovery without recurrence.

## Conclusions

A rare intrascrotal lipoblastoma is seldom made accurate preoperative diagnosis; however, the accurate preoperative suspicion of this tumor leads to the minimal invasive tumorectomy via scrotal skin incision and favorable postoperative recovery without recurrence.

## Consent

Written informed consent was obtained from the parents of the patient for publication of this case report and any accompanying images. A copy of the written consent is available for review by the Editor-in-Chief of this journal.

## Abbreviations

AFP: alpha-fetoprotein
b-hcg: beta-human chorionic gonadotropin
CT: computed tomography
MNTI: melanotic neuroectodermal tumor of infancy
MRI: magnetic resonance imaging
RMS: rhabdomyosarcoma
US: ultrasonography
